# Reproductive health needs of adolescent and young adult women with pediatric rheumatic diseases

**DOI:** 10.1186/s12969-020-00460-7

**Published:** 2020-08-17

**Authors:** Kristine Carandang, Veronica Mruk, Stacy P. Ardoin, Brittany Huynh, Megan E. B. Clowse, Elise D. Berlan, Cuoghi Edens

**Affiliations:** 1 Department of Psychiatry, University of California, La Jolla, San Diego, California USA; 2 Child and Adolescent Services Research Center, 3665 Kearny Villa Rd., Suite 200N, San Diego, California 92123 USA; 3 Nationwide Children’s Hospital, Columbus, OH USA; 4 The Ohio State University, Columbus, OH USA; 5 Indiana University School of Medicine, Indianapolis, IN USA; 6 Duke University Medical Center, Durham, North Carolina USA; 7 University of Chicago, Chicago, IL USA

**Keywords:** Reproductive health, Sexual health, Family planning, Adolescence, Young adulthood, Rheumatic disease, Transitional care, Pediatric rheumatology

## Abstract

**Background:**

The purpose of this study was to identify reproductive health knowledge gaps and topics that concern adolescent and young adult (AYA) women with pediatric rheumatic diseases and their parents.

**Methods:**

Data collection occurred in two cohorts. In the first cohort, young women (15–20 years old) with pediatric-onset rheumatic conditions and their parents were recruited from a single, academic pediatric rheumatology center. In the second cohort, young women (18–25 years old) with pediatric-onset rheumatic conditions were recruited from a national conference for families with pediatric rheumatic diseases. This resulted in 20 adolescents and young adults (18.3 ± 2.4 years old), and 7 parent focus group participants. Focus group leaders facilitated discussions centered on reproductive health topics that participants identified as important, their sources of knowledge, and preferences for patient education and ongoing follow-up. Data were summarized independently by 4 researchers to reduce potential bias and subsequently analyzed using rapid qualitative analysis.

**Results:**

All participants, regardless of diagnosis, medication, current sexual activity, or current intention to have children, expressed concern about the effect of their rheumatic condition and medications on fertility, risks to mother and child during and after pregnancy, and obtaining safe and effective contraception. Additionally, some participants discussed the burden of disease and its potential impact on motherhood. Finally, participants raised concern around the effect of disease and medication on routine reproductive health care, such as menstrual cycles, feminine self-care, and preventive exams. Three themes emerged: 1) participants had been advised to avoid unplanned pregnancy, however reported receiving inadequate explanation to support this instruction, 2) participants conceptualized reproductive health as tied to rheumatic disease management and thus suggested ways to include family members in discussion, and 3) rheumatology practitioners were not considered a resource of reproductive health information.

**Conclusions:**

Young women and their parents reported dissatisfaction with the availability, quantity, and quality of reproductive health information they received, particularly when related to their pediatric-onset rheumatic disease. These findings provide an initial step in understanding the patient perspective of reproductive health in rheumatology, and how to address these concerns in the care of young women with rheumatic diseases.

## Background

The rheumatology community has recently emphasized the reproductive health concerns of women they care for [1, 2], as they are at increased risk for negative pregnancy-related outcomes including pre-eclampsia, prolonged hospitalizations, and maternal and fetal mortality and morbidity [3–5]. Adult women with rheumatic diseases worry about fertility, chances offspring will be affected by their rheumatic disease, and the challenges of pregnancy and motherhood [6, 7]. Little is known about how rheumatology providers address reproductive health issues with their patients, especially among adolescent and young adults (AYAs). While historically advised they should not get pregnant, women with chronic diseases are as sexually active as their peers [8] with women ages 15–19 years old having the highest rate of unintended pregnancy in the United States [9]. Teratogenic medication use is common in this group and use of reliable contraception is low and inconsistent, which increases the risk of adverse pregnancy outcomes [10, 11]. Still, scant data, particularly regarding patient perspectives, are available concerning pregnancy complications and outcomes in AYAs with rheumatic diseases [12, 13].

The aim of this study was to assess awareness of and experience with reproductive health issues among AYAs with rheumatic diseases and their parent(s), with an emphasis on ascertaining knowledge gaps, sources of information about reproductive health, preferences for communicating information, and topics particularly meaningful to participants.

## Methods

### Participants

Participants were recruited in two settings at two different time points. The first cohort was recruited from the rheumatology clinic at a large, academic, pediatric center. English-speaking females (ages 15–20 years) diagnosed with a rheumatic disease and their parents/legal guardians were consecutively recruited during standard-of-care visits. The second cohort consisted of participants recruited through e-mails sent to AYA registrants of a multi-day, annual, patient-focused, educational and family networking rheumatic disease conference (900+ parent, teen, and child registrants). This second cohort included English-speaking females (ages 18–25 years) with self-reported pediatric-onset rheumatic diseases; no parent group was conducted. For each study, methods were approved by the local Institutional Review Board. Informed assent, consent, and parental permission were collected according to age specifications.

### Focus group facilitation guide

Experts in reproductive health, pediatric and adult rheumatology, and adolescent medicine developed a focus group guide (Additional file 1) to prompt discussion exploring reproductive health concerns in relation to rheumatic diseases, sources of reproductive health information, and preferences for sharing this information. This guide was utilized to conduct all focus groups.

### Procedure

For the first cohort, participants were divided into three groups: high school (15–17 year-olds), post-high school (18–20 year-olds), and parents/guardians. Recognizing the differences in cognitive and social development between middle and late adolescence and that the average age of first sexual encounter is 17, groups were chosen to highlight potential differing perspectives, experiences, and preferences between developmental stages. Three focus groups lasted 90 min each in duration, taking place in a private meeting room adjoined to the hospital. Two formally trained, experienced qualitative research facilitators and an internal medicine-pediatric trained rheumatologist observer were present, none of whom had prior relationships with participants. For the second cohort, the focus group was held in a private meeting room and was led by the rheumatologist who observed the first cohort and an additional pediatric resident with qualitative research training. Immediately preceding each focus group, participants completed a questionnaire collecting demographics, diagnoses and medications, and sexual health habits; parent questionnaire mirrored the AYA questionnaire. Focus groups were audio-recorded and transcribed. Transcripts were supplemented by simultaneous field notes.

This study employed rapid qualitative analysis to identify key topics of interest while maintaining methodological rigor [14]. This method produces findings that overlap with the framework method of thematic analysis, while conserving time [15]. Primary qualitative analysis occurred after the first data collection. First, using a primarily deductive lens, a qualitative researcher created a transcript summary for each group by recording observations and pertinent data under pre-determined domains that were identified during the creation of focus group facilitation guides. Next, taking a more inductive approach, the same researcher noted other phenomena, quotes of interest, and reflections that emerged within participants’ discussions [15]. To reduce potential bias, a research assistant re-checked transcripts and summaries ensuring inclusion of all pertinent information. Summary points and corresponding data quotes were transferred onto a data matrix and independently reviewed by three researchers in order to generate memos and group quotes into topics and themes. For the second cohort, the same qualitative researcher and one additional researcher independently created transcript summaries, which were compared to ensure agreement that all key points were included. Summary points were then incorporated into the existing data matrix in order to determine if they confirmed previous themes or introduced alternative perspectives. Quantitative survey data were analyzed using basic percentages and measures of central tendency.

## Results

A total of 20 young women (18.3 ± 2.4 years old) and 7 parents participated across both cohorts. For the first cohort, 18 additional AYAs and 17 additional parents were initially consented in-clinic, however 28 of these (65%) did not attend. Demographic data for AYA participants is presented in Table 1. There were no notable differences between the total sample of AYAs who consented and those who participated. Because the second cohort was consented on-site, no-show rates did not apply. Demographic data for the parent focus group is presented in Table 2.

**Table 1:** Adolescent and young adult participant demographics

	Cohort 1			Cohort 2	
	Consented AYA* (***n*** = 27)	High School (***n*** = 7)	Post High School (***n*** = 2)	Conference Group (***n*** = 11)	All AYA Participants**
Age (years +/− SD)	17.3 ± 1.5	16.2 ± 1.0	18.0 ± 1.4	20.5 ± 2.5	18.3 ± 2.4
**Ethnicity (%)**					
African-American/Black	3 (11%)	1 (14%)			1 (5%)
Asian				1 (9%)	1 (5%)
White	21 (78%)	5 (71%)	2 (100%)	10 (91%)	17 (85%)
Hispanic/Latino	1 (4%)	1 (14%)			1 (5%)
Bi−/ Multi- racial	2 (7%)				
Disease ***					
Juvenile idiopathic arthritis (JIA)	19 (70%)	5 (71%)	1 (50%)	7 (64%)	13 (65%)
Systemic lupus erythematosus/ Mixed connective tissue disease/ Juvenile dermatomyositis	4 (15%)	2 (29%)		3 (27%)	5 (25%)
Other (Specified below)	4 (15%)				2 (10%)
Granulomatosis with polyangiitis	1 (4%)				
Orbital pseudotumor	1 (4%)				
Behçet’s disease	1 (4%)		1 (50%)		
Chronic recurrent multifocal osteomyelitis + JIA				1 (9%)	
Rheumatoid arthritis	1 (4%)				
Medications ***					
Methotrexate (MTX)	3 (11%)	2 (29%)			2 (10%)
Leflunomide (LEF)	2 (8%)	2 (29%)			2 (10%)
Biologic only	7 (26%)			3 (27%)	3 (15%)
Combination (MTX/LEF+ biologic)	9 (33%)			5 (45%) ^^	5 (25%)
Small molecule				1 (9%)	1 (5%)
Mycophenolate	2 (8%)	2 (29%)			2 (10%)
Azathioprine	1 (4%)				
None / No medication listed	3 (11%)	1 (14%)	2 (100%)	2 (18%)	5 (25%)

*All consented AYA for cohort 1 (those that showed and did not show combined)

**Excludes participants that did not show to cohort 1

***Self-reported in cohort 2

^^ One participant of cohort 2 self-reported methotrexate, rituximab, and mycophenolate concurrently and was placed in the combination group

**Table 2:** Parent participant demographics

Age (years) +/− SD	47 ± 5.4
Gender [n (%)]	
Female	6 (86%)
Male	1 (14%)
Highest level of education [n (%)]	
High school	3 (43%)
College	2 (29%)
Post-grad education	2 (29%)
Household income, yearly ($) [n (%)]	
25–49,999	1 (14%)
50–99,999	3 (43%)
> 100,000	3 (43%)
Ethnicity [n (%)]	
White	5 (71%)
African-American/Black	1 (14%)
Hispanic	1 (14%)

Participants’ reported contraception use and sexual activity are depicted in Table 3. Data from high school and post-high school groups were merged to allow for comparison with parents. There were high rates of agreement between AYAs’ and parents’ responses about AYAs’ contraception use and sexual activity. More AYAs in the second, older cohort endorsed having sex (*n* = 7, 63.6%), compared to AYAs in the first cohort (*n* = 1, 11.1%).

**Table 3:** Patient and parent reports of patients’ contraception use and sexual activity

	Cohort 1		Cohort 2
	AYA (*n* = 9)	Parent^a^ (*n* = 7)	AYA (n = 11)
Sexually active [n (%)]			
Yes	1 (11%)	1 (14%)	7 (64%)
No	8 (89%)	6 (86%)	4 (36%)
Contraception use (regardless of sexual activity) [n (%)]			
Yes	5 (56%)	4 (57%)	8 (73%)
No	4 (44%)	3 (43%)	3 (27%)
(Of those who reported contraception) Contraception use by type [n (%)]			
Condoms only	0	0	2 (25%)
Pill, patch, or ring only	4 (80%)	3 (75%)	1 (13%)
IUD or implant only	0	0	2 (25%)
2+ Forms of contraception	1 (20%)	1 (25%)	3 (38%)
Provider who prescribed contraception [n (%)]			
Primary care provider	1 (20%)	2 (50%)	1 (17%)^a^*
Gynecologist	3 (60%)	2 (50%)	5 (83%)
Rheumatologist	1 (20%)	0	0

*Parent answers are regarding their daughter (AYA participant)

Using our deductive template, six reproductive health topics were identified, supported by excerpts, about which participants desired more information (Table 4). Of note, participants in the second cohort articulated more concrete scenarios and reproductive health knowledge gaps in comparison to abstract discussions of focus groups recruited in-clinic, which aided in the delineation of these topics.

**Table 4:** Reproductive health topics that were prominent within focus group discussion

Topic	Quote(s)
Ability to bear children (e.g. effects of medication and disease on fertility; physical ability to have sex and/or safely carry and deliver)	• Post-high school: *My grandma tells me [my blood] has the Rh-negative factor, and that can cause my blood or something to attack the child [fetus]. And it runs in my family and along with that and the immune disorder, I just, I’m afraid [a successful pregnancy] is not going to be able to happen* • High school: *They do discuss some of the long-term effects with me, but not particularly to like reproductive health … [But] I still think about the future … Sometimes I lay in bed and I’m like, I want to have this many kids, I want to name them this, you know? They don’t really talk about that.* • High school: *My hips and knees get, like, really bad, so just thinking about having sex, it would hurt for me, because my legs don’t move in that position … Simply the act of having sex would be really painful.*
Interaction between disease, medications, and maternal outcomes pre- and post-partum (e.g. ability to continue medications, risk of flares)	• Post-high school: *How would pregnancy affect the disorder itself? Like if it would worsen it or not … I know a lot of people who get like gestational diabetes. Would something like that make my immune condition worse kind of thing? It’s not really a thing of getting pregnant, it’s while you’re pregnant- it’s not a short-term thing … If being on this medicine typically makes you go into labor? Just stuff like that that you don’t necessarily think about, and you’re put on it.* • Parent: [*After my daughter temporarily stopped her medications], I saw just how symptomatic she became and how she was unable to manage pain... She and I had a conversation at that time about, “Is this what’s going to happen if I ever want to have kids? Am I going to have to endure this kind of progression of my disease and this kind of pain if I wanted to clean my body out to be able to safely have kids?”* • Conference group: *I have a hip replacement and my surgeon is kinda talking about getting pregnant with that, and was talking about with giving birth it could become dislocated, it could crack, it could do all these things while you’re birth on top of that that could like ruin my actual hip. And I don’t know what outweighs the other.* • Conference group: *I have dermatomyositis and I’m currently in remission. But if, like, if I were to have a kid in like 10 years or whatever, would I trigger a flare?*
Interaction between disease, medications, and fetal outcomes (e.g. heredity of disease, length to wash out medications, birth defects)	• High school: *When you think about how even just getting something like psoriasis can give you like all kinds of other stuff because you’re more susceptible, it’s kind of like scary to think about, like passing that on. Because it is hard to deal with all of the issues and like getting to so many appointments and stuff. It’s kind of hard to think about, putting that on somebody else too.* • Conference group: *What’s the chance for our kid getting this because, I mean, we make the best of it but it sucks. I don’t want my kid to have this life … I’m at that point, like, I don’t wanna have kids if they’re gonna have this because I feel like this is terrible. We do what we can with it but like, it sucks. So like I just wanna know what the chances are I guess.*
Obtaining safe, effective contraception	• High school: *[Compared to my friends], it’s kind of a big deal for me because I can’t go to a pharmacy and like pick out birth control … but I kind of have to go further and like talk to my doctors, talk to my parents, and then I don’t know, figure it out more, see what’s, what is more safer for me.* • Parent: *For my daughter, the doctor prescribing those birth control pills is not her rheumatologist, you know, someone who certainly knows her history, but who isn’t a specialist in that area, and so is there the level of trust that: Does the GP have the knowledge base to know that these are a good idea or not a good idea [given her rheumatology medications]? and I should point her in some other direction?*
Effects of disease, medications on routine reproductive healthcare (e.g. managing menstrual cycle, obtaining HPV vaccine)	• High school: *We know what tampons and pads are and stuff like that, but like with my limited movement and stuff, sometimes it’s hard to like take care of myself in a way that’s like good for my body and stuff … Schools aren’t necessarily the best at like telling everybody how to properly take care of yourself.* • Conference group: *I could list my medicines off, but can that mess with your menstrual cycle?* • Conference group: *[After discussion on HPV], being on immunosuppressants, are we at a higher risk for things like cervical cancer?*
Ability to take care of a child (e.g. Need a supportive partner to share the load) *Not discussed at conference-focus group	• Post-high school: *The symptoms that I get are so bad that I feel like if I were to tell my partner or something that they wouldn’t understand and they will just automatically think that there is something wrong with me, like sexually wrong with me, and there isn’t but it’s hard to talk about when you get symptoms like that.* • Parent: *All the big picture sort of decisions that go along with having a daughter who very much wants to be a mother. I mean, [my daughter] has been that girl who babysat everybody’s babies, and I know that’s something that she really will want in her life … What is that going to mean for her disease? And for her whole family structure? And yeah, will she have a partner who gets it?*

Taking an inductive and exploratory lens, three themes were identified as described below.

1. **Directives without explanation:** Across all focus groups, participants reported that AYA patients were instructed by their rheumatology providers to avoid pregnancy when taking teratogenic medications but received little other information about the relationship between their rheumatic disease and pregnancy, nor further discussion on pregnancy prevention. Some participants recalled being told about potential side effects when teratogenic medications were prescribed, though others did not recall receiving this information. One high school participant stated: “*No one’s ever talked to me about what the side effects of taking these drugs could be like in the long run … it would be nice to know if taking [specific medications] has any effect on my reproductive system years later*.” Without more in-depth information, AYAs and parents held a vague understanding around AYAs’ ability to become pregnant, safely carry and deliver a baby in the future. Participants expected rheumatologists to provide situational advice rather than being able to currently plan: “*If I plan on getting pregnant, I have to talk to [my rheumatology providers] because it could affect the baby.*” or “*If I did get pregnant, I had to call [my rheumatology providers], they had to talk about it and see if I could even continue the pregnancy*” (two high school participants). In addition, AYAs who were already having sex without the intention of becoming pregnant had limited information about contraception: “*No one ever talks about how do you not [have kids] besides, ‘Don’t have sex, don’t get pregnant, don’t die”* (conference group participant).

Regardless of current sexual activity or pregnancy intention, all participants desired a deeper understanding about how their rheumatic disease affects sexuality and future reproduction (see Table 4): “*If you want to have children, you want to make sure everything’s okay; but even if you’re not to that point, you still yourself want to be okay*” (post-high school participant). AYAs and parents lacked awareness and expressed deep concern about three topics related to family planning: possible infertility, potential negative pregnancy outcomes for AYAs and their offspring, and anticipated hardships during motherhood. All participants assumed they would need to stop all of their medications while pregnant/breastfeeding. Additionally, participants desired information about how rheumatic diseases interact with their reproductive organs and potential offsprings’ risk of developing a rheumatic disease.

Rheumatology providers’ involvement in providing reproductive health education was deemed important, although lacking in all cohorts, and participants suggested ways to improve communication dynamics to allow AYAs to broach these topics comfortably. AYAs discussed the potential usefulness of detailed pamphlets and/or recommended websites.

*“I wish I would have gotten a pamphlet that said “Reproductive Health” that really explained … here are the common medications you might have for JIA, which ones could affect this, and which ones long-term. I think that would have been really nice so I can look back as I get older”* (high school participant).

*“If [rheumatologists or drug companies] had a whole pamphlet dedicated to like women’s health and like fertility, pregnancy, like risks, complications, and all of that”* (conference group participant).

Some AYAs and parents wanted the opportunity to have a thorough annual discussion wherein healthcare providers prompted reproductive health concerns that families should consider. One high school participant wanted providers to *“confirm that I still understand what’s going on because now I am older [than 8 years old when I was diagnosed] and can think about [pregnancy] more and know the actual side effects instead of just like, ‘by the way, don’t do this in the future.’”* Participants suggested this discussion be built into the electronic medical record system. Across all formats, the ultimate goal of education was normalization: “*Patients have questions, but they don’t want to ask them. So let’s more normalize at a young age and you know when that time comes, you know, we’re more open to talk about it*” (conference group participant).

2. **Altered discussion around sex and intimacy:** Across groups, participants commented on the communication dynamics between AYAs, parents, and healthcare providers when discussing reproductive health concerns. Participants viewed decisions around reproductive health, such as taking contraception or having sex, as inextricably tied to AYAs’ ability to manage their symptoms and maintain their health. As a result, participants agreed that parents/guardians should be included within some of this discussion, but opinions varied on how parent involvement should be implemented.

In the first cohort, AYAs’ families had grown accustomed to speaking openly about sexual activity, menstrual periods, and contraception use, both within and outside of healthcare visits, and therefore AYAs erred on the side of openness and honesty with family members (e.g. mother, father, grandmother). One high school participant stated, *“It makes [sex] more a public thing, [rather] than a private matter between two people.”* First cohort participants noted closer family relationships may have developed after overcoming a difficult rheumatic disease course and/or needing to collaborate to obtain sufficient health-related information.

Both AYAs and parents acknowledged that some discussions did not come naturally, “*because, you know, it’s still your [parent], you’re still young, it’s still weird.”* For example, several high school participants reported their fathers accompanied them to their healthcare appointments. Despite these participants’ preferences to discuss reproductive health issues in the presence of female guardians, they ultimately deferred to their fathers because of availability:

*“I’d rather my dad know that I had sex and be a little bit uncomfortable with me for a second or upset or whatever he’s going to be, than me being in worse pain or dying or whatever is going to happen because I didn’t tell them that this happened. [My doctors] have to know about it”* (high school participant).

One father participant gave an example of the “*awkwardness*” he felt after being asked to step out of the room in the middle of his daughter’s appointment:

*“The physician asked us [parents] to step outside, and [my daughter] goes, ‘I know what’s coming.’ And I was like, “So do I.” So I awkwardly walk outside while they’re having the conversation [about sexual history]. They’re almost like it was taboo for us to have even talked about it before, and I understand respecting privacy, I mean I completely understand that, but … to presume somehow we’d not had that conversation.”*

Instead of asking parents to step out of the room, this father suggested that physicians consider using techniques that foster communication between daughter and parents rather than “*setting a barrier between them*.”

While the second cohort focus group did not generate the same extent of discussion around this issue, these participants did acknowledge their parents’ role in reproductive health decision making. It was unclear if thoughts about parents’ role stemmed from AYAs’ preferences or their perception of their parents’ preferences: “*I think [parents] would rather have you be safe and effective with what you were trying to do than have you go learn about it from some website in like the dark internet or something. So I think it’s important to talk to parents and like not kick them out of the room all the time during these talks and actually involve them in this decision making.”* Participants commented that some topics (e.g. medications’ potential side effects on reproductive health,) warranted the need for parents’ input more than others (e.g. current sexual activity), and therefore called for a more AYA-centered approach of when to include family within conversations.

3. **Rheumatology providers were not considered a resource of reproductive health information:** *“It’s not really something I guess I think of when I go to rheumatology”* (high-school participant).

Participants reported varied sources of reproductive health information related to rheumatic diseases, but no group consistently reported rheumatology providers as a sought out or trusted resource. High school participants reported limited, if any, discussions with any healthcare provider beyond recommendations not to become pregnant. Within the post-high school group and conference group, a majority of participants reported discussing their reproductive health concerns, including finding birth control options that relieved symptoms that stemmed from their rheumatic disease, with gynecologists. Post-high school participants considered time, clinic flow, and priority on disease management as potential barriers to reproductive health education within rheumatology appointments. Parents mentioned a range of healthcare providers with whom they discussed their child’s reproductive health: primary care providers, fertility specialists, pharmacists, gynecologists, and rheumatologists. Parents were frustrated by the lack of consistency in access to reproductive health experts; for example, one parent described fertility specialists as a “*ghost operation*” that the family was unable to contact when questions arose. In lieu of expert advice, mothers reported drawing on similar personal and female-family member experiences to aid their daughters.

In circumstances when rheumatologists were included in reproductive health discussions, participants reported receiving conflicting information from providers and struggled to determine the best guidance for themselves and their daughters. When discussing medication, family planning, and even which type of provider should be the primary source of advice, participants had the following contributions:

*“My PCP is like almost afraid to treat reproductive health. Because I was asking about like birth control, not like as a contraceptive but just like to regulate my cycle, and she was like, well I don’t know what XYZ of birth control would do with your [rheumatology] meds, so refer to your rheumatologist. My rheumatologist was like, well, you need to see an OB/GYN. And it’s just like, kinda of frustrating”* (conference group participant).

*“I mean, I have a primary care doctor tell me like that I would never be able to get pregnant just from like not taking very much methotrexate, and then my rheumatologist was like, That’s not true at all, so I feel like it’s hard for primary care providers who don’t see our rheumatic conditions that often to like understand the doses of the medications we’re on and like what they’re used for and that they’re different than like using it as a chemotherapeutic agent”* (conference group participant).

Within the parent group, participants shared potential strategies to overcome these potential discrepancies (e.g. using the patient portal to coordinate advice).

In order to fill reproductive health knowledge gaps, AYA groups reported turning to family members for advice and consulting the internet. Young women used this resource primarily to research medication side effects and brainstorm questions, topics, and/or concerns to discuss at clinic visits. All groups qualified their internet sources as “*reliable*” but were unable to describe how sites were vetted.

## Discussion

The purpose of this exploratory study was to address knowledge gaps in reproductive health awareness and education from the perspective of AYA women with pediatric-onset rheumatic diseases and their parents. Participants identified six topics about which they wanted more information (Table 3). In addition, findings from this study identified three themes through analysis of transcripts. First, participants were told to avoid pregnancy while on teratogenic medications but were rarely provided resources or information about reproductive health risks or future pregnancy and family planning. Second, reproductive health was inextricably tied to AYAs’ rheumatic disease management, and therefore parent inclusion within reproductive health decision making was normalized. As AYAs matured, discussions became more complicated when trying to respect both patient autonomy and privacy. Third, rheumatology providers were not considered a prominent resource for reproductive health information, even when topics directly related to their rheumatic diagnosis or medications.

In spite of the unmet facet of clinical care reported almost two decades ago [16], AYAs and their parents continue to report dissatisfaction as to how reproductive health is addressed in pediatric rheumatology clinics [17]. Our findings mirror published literature reporting a dearth of reproductive health information available to rheumatology patients of child-bearing age [5, 6, 18], and young adults with other chronic diseases [19]. In other studies, women reported making key decisions about reproductive health based on misinformation, anxiety, and fear versus medical input and reliable resources [6, 7]. Similarly, our participants, who had a broad spectrum of diagnoses and medications used, voiced that they had insufficient information to make informed decisions about future pregnancy and family planning, and did not know where or from whom to obtain the most accurate advice. While patients with specific diagnoses that convey high risk (e.g. systemic lupus erythematosus with antiphospholipid positivity) should receive specific pregnancy counseling, it remains telling that all focus group participants wanted more information about reproductive health. Some participants with mild juvenile arthritis even questioned their ability to have children at all. These issues highlight a critical disconnect between patients’ and clinicians’ priorities and knowledge: reproductive health is not yet routinely addressed as part of transitional care [20] and ranks low on clinicians’ research priorities [21]. Reasons why reproductive health inquiry and counseling has not become standard in pediatric subspecialty care is likely multifactorial and is a topic of ongoing research. Adult rheumatologists have identified time, lack of knowledge and experience, and anxiety surrounding topic as barriers to family planning counseling and reproductive health care [22].

Our results assert age-specific needs and timely exploration of relationships and sexuality amidst the transition from adolescence to adulthood in rheumatic disease patients. Regardless of reported sexual activity, a majority of participating young women were using contraception and all wanted anticipatory reproductive health guidance from their rheumatology providers. These patient perspectives are congruent with existing literature that highlight the need to discuss sexual health topics within rheumatology transitional care [23, 24]. To aid in these discussions, adolescent psychosocial screening tools, such as HEADSS, have been incorporated into some adolescent and primary care clinics, however, to our knowledge, are used rarely in rheumatology [25, 26]. Over a decade after these gaps were identified [23], training is needed to improve rheumatology provider confidence in discussing reproductive health with AYAs [20] and elevate the role of rheumatologists regarding this topic.

Participants conceptualized reproductive health as tied to their overall disease management, and therefore raised questions about how to include parents within these reproductive health discussions. Unlike other reports [27], younger cohort participants minimized confidentiality concerns around sexual activity and instead wanted consistent, reliable sources of information that could be discussed openly among family members. On the contrary, older cohort participants discussed benefits for parents to be included within some parts of reproductive health decision making (e.g. medications and reproductive health side effects) and allow for privacy at others (e.g. current sexual activity). These points may have several explanations. AYAs and parents of the first cohort, who simultaneously attend a reproductive health focus group, were likely to be more comfortable with the subject and may emphasize confidentiality less than those who declined participation and those in the general population. Most of these young women were not engaged in sexual activity, which may have increased their willingness for open family discussion. Second cohort participants were older, did not require parental permission to participate, and were more sexually active. This latter group also self-selected to participate in the focus group at a national patient-centered conference, suggesting a higher level of engagement. Young women with pediatric-onset rheumatic diseases may have heterodox ideas surrounding confidentiality given their unique healthcare experiences. Our findings offer valuable insight when considering how to implement existing communication guidelines, confidentiality policies, and shared decision making with AYAs and parents around reproductive health [28] and warrant further scholarly exploration.

Limitations of this study include a small sample size and demographically similar participants. AYAs are a hard-to-reach population for both clinical appointments and research endeavors [29]. While the first cohort may contain bias due to recruitment from a single academic center and unexplainable high dropout rate, we attempted to mitigate this bias by recruiting through a patient-centered conference; attendees forming the second cohort received their care from a variety of rheumatic practices across the country. Focus groups have an inherent bias based on persons who choose to participate. Due to lack of participant diversity, the reported findings may not be generalizable to other dimensions of identity, such as race/ethnicity, culture, and religion, that may intersect with patients’ preferences for sexual and reproductive health information, as well as specific rheumatic diseases and practice settings. New perspectives continued to be presented in our final focus group; therefore, data saturation was not met for these topics. However, as the purpose of focus groups is to generate ideas for further research and intervention rather than producing generalizable results, these findings remain applicable and relevant in highlighting the valid concerns, knowledge gaps, and preferences of patients. Given this study’s limited scope and desire for subject candor, we did not include male AYAs or their parents, an already overlooked population in reproductive health literature who may have similar concerns around medication side effects, heredity, and fertility.

## Conclusions

This exploratory study was conducted to identify AYAs’ reproductive health needs and to better understand patients’ and parents’ perspectives. Young women and their families see a critical need to discuss reproductive health concerns with their rheumatology providers and desire more topic-specific information. Potential future steps include additional qualitative assessment of patient, family, and provider experiences; barriers to patient education; and recommendations for developing tools that enable productive and timely conversations about reproductive health amongst pediatric rheumatology care providers, patients, and family members.

## Supplementary information

**Additional file 1.** Focus Group Facilitation Guide for AYAs: Main questions only

## Data Availability

The datasets generated and analysed during the current study are not publicly available per the restrictions of the approved study protocol for qualitative data.

## Abbreviation

AYA: adolescent and young adult
