# Supplementary material for: Reproductive health needs of adolescent and young adult women with pediatric rheumatic diseases
Source: Pediatr Rheumatol Online J. 2020 Aug 17;18:66. doi: 10.1186/s12969-020-00460-7 (PMC7433038; doi:10.1186/s12969-020-00460-7)
Supplement: Supplementary file 1 — Additional file 1. Focus Group Facilitation Guide for AYAs: Main questions only [file 12969_2020_460_MOESM1_ESM.docx]

Appendix A. Focus Group Facilitation Guide for AYAs: Main questions only

1. Where do you usually get information about your rheumatic disease?
2. What does reproductive health mean to you?
3. What are some issues you have thought about when it comes to reproductive health and being diagnosed with a rheumatic disease?

- Additional prompts about medications, future family, dating, sex

1. Where have you been able to get the information you know?
   1. Tell us about those experiences.
   - Additional prompts about specific sources (e.g. healthcare providers, parents, Internet, friends/peers) and dynamics within clinic visits
   1. What are some topics you would have liked to talk about but have not been able to?
2. If there were an ideal place to learn about reproductive health and rheumatic disease, what would that look like?

*Questions on *Focus Group Facilitation Guide for Parents of AYAs* mirrored the questions above, asking about “your daughter’s rheumatic disease” instead of “your rheumatic disease.”
